# Identifying key aspects to enhance predictive modeling for early identification of schistosomiasis hotspots to guide mass drug administration

**DOI:** 10.1371/journal.pntd.0013315

**Published:** 2025-07-16

**Authors:** Yewen Chen, Fangzhi Luo, Leonardo Martinez, Susan Jiang, Ye Shen

**Affiliations:** 1 Department of Epidemiology and Biostatistics, College of Public Health, University of Georgia, Athens, Georgia, United States of America; 2 Department of Epidemiology, School of Public Health, Boston University, Boston, Massachusetts, United States of America; Xuzhou Medical University, CHINA

## Abstract

**Background:**

Schistosomiasis, a neglected tropical parasitic disease, threatens the lives of over 250 million people worldwide. In schistosomiasis prevention, high-transmission areas that do not respond to treatments, known as hotspots, pose extreme challenges to the elimination of the disease. Accurate and early identification of such hotspots is crucial for timely intervention, but this is hindered by the limited availability of effective prediction methods.

**Methods:**

Based on the Schistosomiasis Consortium for Operational Research and Evaluation (SCORE) project over a 5-year period, this study developed prediction methods for the first (baseline) year to identify hotspots. Three key aspects were considered: (i) collecting secondary data from public sources to complement baseline schistosomiasis infection data and constructing spatially weighted predictors to incorporate neighboring information; (ii) categorizing predictors to mitigate overfitting and quantifying the importance of each category in hotspot predictions; and (iii) investigating the hotspot imbalance distribution and addressing the imbalance with a sampling-based technique to improve prediction performance.

**Results:**

Compared to the approach using only baseline infection data, the spatially weighted data fusion method achieved relative improvements (RIs) in hotspot prediction accuracy by fusing baseline infection data with each predictor category: 10% with biology, 8.6% with geography, 6.6% with society, 3.5% with baseline infection data around villages, 3.3% with environment, 1.8% with agriculture, and 7.2% with all predictors. Furthermore, across the same predictor combinations, applying the sampling-based technique with the proposed method yielded RIs of 6.5%-37.9%, compared to the approach that did not address the imbalance.

**Conclusion:**

Spatially weighted data fusion using secondary data improved the early identification of schistosomiasis hotspots. Addressing the imbalance of hotspots can further improve the early identification of the hotspots.

## Author summary

Schistosomiasis infection is a major public health problem. The related study shows that mass drug administration (MDA), a widely used method for achieving preventive chemotherapy with praziquantel (PZQ), does not prevent reinfection and the formation of high-risk areas (i.e., hotspots) between MDA rounds. Especially in endemic regions, multiple rounds of MDA are typically required for the elimination of schistosomiasis. This study aims to develop prediction methods that identify hotspots before the first MDA round (i.e., early identification) to guide subsequent treatment efforts. Accurate and early identification of hotspots, however, faces challenges due to a lack of sufficient infection data. Furthermore, the ratio of hotspots to non-hotspots is often highly imbalanced, making it even more difficult to extract useful information from the available baseline infection data to identify these hotspots. To overcome these challenges, we collected secondary data from public sources, applied spatial weighting techniques to construct predictors, and employed synthetic sampling-based methods to mitigate hotspots imbalance. We then developed statistical and machine learning models for hotspot prediction. Our method supports MDA efforts, contributes to schistosomiasis elimination, and improves public health.

## Introduction

Schistosomiasis, a neglected tropical parasitic disease caused by *Schistosoma* trematodes infection, inflicts severe harm on humans, including periportal fibrosis, gastrointestinal symptoms, impaired growth, anemia, and others [1–5]. This disease is widely distributed in Africa, South America, and Asia, predominantly in rural areas with poor socioeconomic conditions [6–8], with more than 70 countries reporting cases [9]. In 2021 alone, at least 251 million people required preventive treatment [10]. In preventive chemotherapy programs, mass drug administration (MDA) with praziquantel is widely used to control and eliminate schistosomiasis [11]. However, despite years of MDA treatments, some high-risk areas remain endemic due to persistent reinfection [12]. In literature, such high-risk areas are referred to as hotspots, particularly persistent hotspots (PHSs). PHSs are defined based on limited reduction in infection prevalence and/or intensity from the baseline year to a given year following MDA [13–17]. These PHSs pose significant challenges to schistosomiasis elimination efforts. The 2022 World Health Organization (WHO) guidelines recommended more frequent preventive treatment in hotspot areas [9], which can contribute to the achievement of a new roadmap, namely, the elimination of schistosomiasis as a public health problem by 2030 [18].

Given this background, being able to make accurate predictions of PHSs, particularly before the first round of MDA, becomes potentially valuable for optimizing treatment strategies, dynamically adjusting preventive resources, and eliminating schistosomiasis in a timely manner. However, prior work on predicting PHSs typically required waiting for at least three years of infection data to achieve sufficiently accurate predictions [14]. More recently, an early identification method has been developed to predict hotspots in the baseline year by combining data on schistosomiasis infection with geospatial secondary datasets [17]. However, accurate and early identification of hotspots remains a significant challenge. This is because (1) the performance of prediction models is often influenced by the construction and combination of predictors from different domains, and (2) the distribution of hotspots themselves. These aspects have not been thoroughly investigated in previous studies.

To advance the accurate and early identification of schistosomiasis hotspots, this study hypothesizes that incorporating spatial correlation in the construction of predictors and/or accounting for the underlying distribution of hotspots in the developement of predictive models will enhance early identification of hotspots. The rationale is that safe water, hygiene practices, and occupational exposure risks are usually linked to schistosomiasis transmission, and geographically proximate villages often share similar risk factors [9,19,20]. We test this hypothesis by investigating several key aspects critical to the development of predictive models: (i) analyzing the spatial correlation and heterogeneity of the disease and applying spatial weighting methods to summarize secondary spatial grid level data from publicly available sources to the villages as predictors; (ii) categorizing the resulting spatially weighted predictors, combining each predictor category with baseline infection data of schistosomiasis to develop prediction models, and assessing relative improvements (RIs) to quantify the importance of each category in hotspot prediction, compared to models using only baseline infection data; (iii) enriching the model pool, particularly by incorporating models that account for the nonlinear nature and complex correlations of the disease; and (iv) investigating the hotspot imbalance, where the proportion of hotspots is often much lower or higher than 0.5, and applying a sampling-based method to mitigate its impact on the accuracy of prediction models. These aspects were not taken into account in previous approaches.

**Fig 1:**
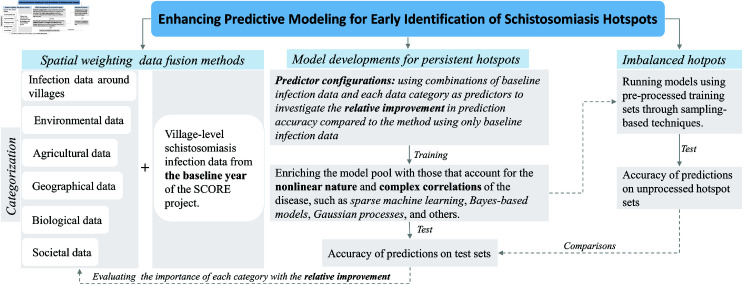
Study framework for enhancing early identification of schistosomiasis hotspots (early identification refers to the use of infection data only from the first year to develop prediction models).

Based on the work above, we developed statistical modeling methods to improve the early identification of schistosomiasis hotspots. The main framework of the present study was summarized in Fig 1.

## Methods

### Study population and outcomes

**Fig 2:**
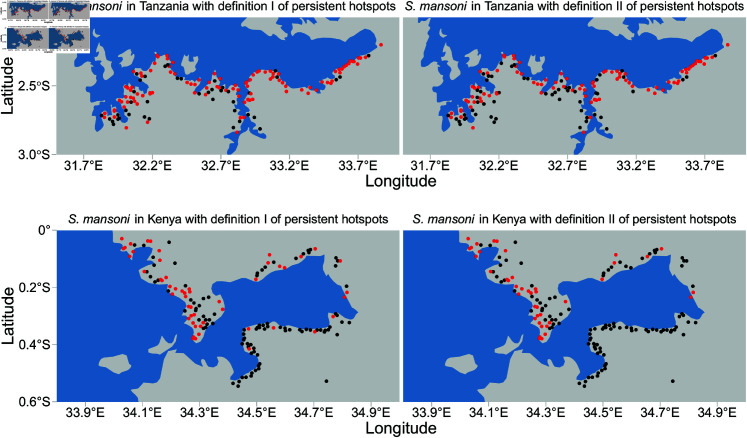
Map of hotspot areas around Lake Victoria, highlighted in red points, in Tanzania (top) and Kenya (bottom) using persistent hotspot definitions I–II. The map layers were created using publicly available world map data from Natural Earth, accessed via the R package *rnaturalearth* [24].

This study focused on *Schistosoma mansoni* (*S. mansoni*) infection hotspots in villages within the Lake Victoria basin, including 147 villages in Kenya and 148 villages in Tanzania (Fig 2). *Schistosoma* infection data in these villages were obtained based on a randomized controlled trial from 2011 to 2015 through the SCORE project [21]. In the SCORE project, the 295 study villages were randomly assigned to six arms, with each arm annually receiving one of three types of treatments (school-based treatment, community-wide treatment, or no treatment) through mass drug administration (MDA) with praziquantel from 2011 to 2015 (S1 Fig) [22]. Disease-related data were collected through annual epidemiological surveys, either shortly before or during MDA programs [14]. We considered two main outputs of the surveys: prevalence and intensity of infection among children aged 9 to 12 years (S1 Text). In addition, both outputs were used to define persistent hotspots (PHS) using the following two methods:

- *PHS definition I*: Included villages with a relative reduction in prevalence of less than 35% or a relative reduction in intensity of less than 50% from baseline to Year 5 [14,15,23];
- *PHS definition II*: Included villages with less than a 35% relative reduction in prevalence from baseline to Year 5 and a greater overall prevalence than 10% in Year 5 [9,17].

Such PHSs, according to each definition, were presented in Fig 2, and the proportion of PHSs was provided in S1 Table. These PHSs were then used as study outcomes. Our aim was to develop methods for the early identification of PHSs in the first year (referred to as baseline methods). The advantage of the baseline method is that it does not require *Schistosoma* infection data after the baseline.

### Exploring patterns of schistosomiasis, spatially weighted data fusion, and categorizing datasets

In this study, we employed empirical variograms, a standard geostatistical tool used to understand spatial structure and detect spatial patterns in infection prevalence and intensity, illustrating how infection data vary and correlate across space [25]. To account for spatial correlation at short distances while filtering out redundant noise from longer distances (Sect 3.1), a spatially truncated inverse distance weighting (spTIDW) approach was applied to each village to produce spatially weighted prevalence and intensity predictors [26,27]. For simplicity, we provided a category label with *infection data around villages* for the resulting predictors, which were subsequently used to explain whether the village was a hotspot or not.

Moreover, incorporating a broader range of schistosomiasis-related variables from different domains usually helps enhance the performance of prediction models. However, large number of predictors may increase the risk of overfitting, resulting in poor out-of-sample performance. Categorizing the variables by domain can help manage model complexity, provide deeper insights into hotspot formation, and improve the interpretability of predictive models. We used a knowledge-driven method to categorize these secondary predictors into five distinct categories: *environment*, *agriculture*, *biology*, *society*, and *geography* (S2 Text). For each category, we collected secondary datasets from publicly available sources based on prior studies [9,17,18,28,29]. Most of datasets are defined on spatial regular grids (S2 Table), and we applied spTIDW to summarize them from the grid level to the villages to fuse the baseline infection data. The resulting spatially weighted predictors were used to account for the hotspot status of the villages, similarly to those from *infection data around villages*. Some of these predictors have been shown to be closely associated with the status of schistosomiasis hotspots, although the strength and direction of their relationships may vary across the study villages [28].

### Developing prediction models using different combinations of predictors

We investigated eight predictor configurations to develop prediction models: (C1) only baseline infection data, (C2) baseline data and *infection data around villages*, (C3) baseline data and *environment* predictors, (C4) baseline data and *agriculture* predictors, (C5) baseline data and *biology* predictors, (C6) baseline data and *society* predictors, (C7) baseline data and *geography* predictors, and (C8) all predictors. In light of this, relative improvements (RIs) of each predictor configuration from (C2) to (C8) can be assessed in terms of prediction accuracy by comparing them with the method developed using (C1) (see Sect 2.5 for the definition of RI). Therefore, the resulting RIs allow for quantifying each category’s importance in hotspot predictions.

From a modeling perspective, hotspot prediction is a classification problem with two classes. We tested fourteen classification models for each of the eight configurations, eight of which had previously been used to predict hotspots [14,17]. These included GBM (gradient boosting machine), RF (random forest), Tree (a single decision tree), Logit (elastic-net logistic regression), LASSO (logistic regression with the least absolute shrinkage and selection operator), LGT (traditional logistic regression), SVM (support vector machine), and an ensemble of these models implemented using the R package h2o [30]. To better account for the nonlinear nature of hotspot formation and the complex correlations of the disease, six additional models were investigated through this study. These models included LogitGPs (logistic regression with Gaussian processes [31]), sparseSVM (regularized SVM [32]), DyTrees (dynamic tree models implemented by particle learning [33]), regLogit (regularized logistic regression based on Gibbs sampling schemes [34]), Probit (probit regression [35]), and DNN (deep neural network model [36]).

### Improving prediction models through random oversampling

In addition to considering different predictor configurations and developing more flexible prediction models, we also investigated the impact of the imbalance proportion between hotspots and non-hotspots on prediction accuracy. From an application perspective, an imbalanced proportion can heavily affect the development of prediction models, as such patterns often cause the models to learn features primarily from one side of either hotspots or non-hotspots, depending primarily on which has higher proportion, i.e., the imbalance learning problem [37]. This often results in low accuracy in hotspot predictions. To mitigate the influence of imbalanced hotspots on the accuracy of prediction models, a synthetic sampling-based method was applied for each of the eight predictor configurations mentioned earlier. This involved using current training data $T_{n}=\{(x_{i},y_{i}):i=1,\ldots ,n\}$ to generate new synthetic training data $T_{m}=\{(x_{k}^{*},y_{k}^{*}):k=1,\ldots ,m\}$ through random oversampling, where $x_{i}$ represent the predictors of the village *i*, the outcome $y_{i}\in \{{𝒴}_{0},{𝒴}_{1}\}$, and here ${𝒴}_{0}=0$ (non-hotspot) and ${𝒴}_{1}=1$ (hotspot). This procedure includes three steps for each *k*:

(a) Randomly select $y_{k}^{*}={𝒴}_{j}$ with the probability $\pi_{j}$ for *j* = 0 or 1;
(b) Randomly select $(x_{i},y_{i})\in T_{n}$, such that $y_{i}=y_{k}^{*}$, with the probability $\frac{1}{n_{j}}$, where *n*_*j*_ is the number of ${𝒴}_{j}$ within *T*_*n*_; and
(c) Sample predictors $x_{k}$ from $K_{H_{j}}(\cdot ,x_{i})$, with $K_{H_{j}}$ being a probability distribution, depending on the smoothing matrix $H_{j}$; see [37] for more details on setting $K_{H}$ and H.

We implemented this method using the R package ROSE [38]. Following common practice, we set $\pi_{j}=0.5$ and chose a synthetic sample size of *m* = 300, which was comparable to the village size of *n* = 295. To further illustrate the benefits of addressing unbalanced problems for improving hotspot prediction, synthetic minority over-sampling (SMOT) was also implemented using the R package performanceEstimation [39]. To reduce the risk of overfitting, these data synthesis methods were applied only to the training set, after data splitting in cross-validation (CV; Sect 2.5) [40].

### Cross-validation methods and evaluation criteria

Three scenarios were considered in model development and validation: within-country, combined-countries, and between-countries. CV was performed to validate the performance of the prediction models in each scenario. The within-country scenario included villages only from a single country in each CV setting. This scenario involved two CV settings. The first CV setting consisted of villages only from Kenya, while the second contained villages only from Tanzania. The combined-countries scenario involved a single CV setting, where the villages of both countries were completely incorporated. The scenario between countries involved two CV settings, labeled as *Between I* and *Between II*. In *Between I*, the training set consisted of villages only from Kenya, while the test set included villages only from Tanzania. In contrast, in *Between II*, the training set contained villages only from Tanzania, while the test set included villages only from Kenya.

In each CV setting of the within-country and combined-countries scenarios, 70% of the villages were randomly selected as the training set for model development, while the remaining 30% were reserved as the test set for model evaluation. In each CV setting of the between-countries scenario, 70% of the villages of the training set were randomly chosen to develop the models, while all the villages of the test set were used to evaluate the performance of the models in predicting the hotspots.

This work used the accuracy of the predictions on the test set as the evaluation metric. Accuracy in each simulation was measured as the proportion of correctly predicted hotspots and non-hotspots out of the total number of both. We conducted 200 simulations for each CV setting and calculated the average accuracy of the model across simulations. To evaluate the improvements of the proposed data fusion method with respect to other approaches, we further let $\text{RI}=\frac{\text{Acc II}-\text{Acc I}}{\text{Acc I}}\;\times \;100$, where Acc I and Acc II refer to the average accuracy of the reference approach and the proposed method for a specific model, respectively.

## Results

### Developments of spatially weighted predictors

Empirical variograms showed that the spatial correlation ranges for prevalence in Kenya and Tanzania were approximately 12 km and 50 km, respectively, while for intensity, the spatial ranges were 20 km and 40 km in Kenya and Tanzania (S3 Fig). In addition, the disease exhibited spatially heterogeneous characteristics, with significant differences in disease prevalence and/or intensity between two villages located very close to each other, resulting in one being a hotspot and the other not (Fig 2). In this case, using commonly employed methods, such as nearest neighbor, to downscale secondary spatial grid data can result in the same value for the resulting predictor in two villages that are very close to each other. This is because these two villages may share the same closest spatial grid to derive predictor values. In contrast, spTIDW utilized multiple grids to construct spatially weighted predictors, typically resulting in different predictor values. This can be beneficial in identifying differences caused by small-scale variations. This work used a 20 km correlation range as the threshold in spTIDW to select neighboring villages to construct spatially weighted prevalence and intensity predictors in *infection data around villages*. For spatial grid data, a 50 km threshold was applied to select grids to generate all other spatially weighted predictors from the remaining five categories: *environment*, *agriculture*, *geography*, *biology*, and *society* (see S2 Text and S2 Table for more details of these predictors).

### Spatially weighted data fusion methods for predicting persistent hotspots with definition I

For each model, we calculated RIs of the data fusion method for each scenario and predictor configuration, based on the prediction accuracy detailed in S3 Table. These RIs were then averaged across three CV scenarios for each predictor configuration, resulting in the average RIs (ARIs) presented in Table 1. Compared to models that used baseline infection data only, the fourteen models that employed the data fusion method in most cases achieved positive ARIs.

**Table 1:** Average relative improvements (ARIs, %) for each model in prediction accuracy on test sets from the proposed spatially weighted method using seven different predictor configurations C2–C8, compared to the method using the baseline infection data only (C1).

Combining baseline infection data	Average RIs across the CV scenarios (%)													
With additional predictors	GBM	RF	Tree	Logit	LASSO	LGT	SVM	Ensemble	Logit GPs	sparse SVM	dyna Trees	reg Logit	Probit	DNN
Infection around^1^ (C2)	0.52	4.28	0.18	8.96	10.09	8.06	2.83	5.14	1.99	0.63	0.93	2.41	−0.12	9.04
Environment (C3)	−0.37	10.41	3.82	1.94	2.07	1.30	−2.81	2.38	−3.36	2.41	3.79	−0.57	−1.04	4.89
Agriculture (C4)	−1.79	7.89	1.29	1.76	2.24	−0.02	2.80	−0.27	−3.54	3.09	4.64	1.89	5.11	5.29
Geography (C5)	3.01	11.63	5.19	21.77	21.46	22.53	16.23	15.07	12.35	16.72	7.32	24.26	7.37	26.23
Biology (C6)	1.70	9.98	0.18	20.90	20.10	20.67	9.67	10.30	6.85	7.58	5.65	18.41	4.59	27.92
Society (C7)	−2.69	8.70	3.11	15.93	15.98	20.43	14.74	13.02	8.30	12.86	0.74	19.69	−2.66	18.48
All (C8)	−4.75	12.14	5.61	6.57	6.66	10.61	7.16	2.76	−4.31	10.23	3.91	12.69	2.38	12.01

^1^Infection data around villages (similarly hereinafter).

For each scenario and predictor configuration (S3 Table), we further calculated the RIs from the best of the 14 models, in terms of the highest prediction accuracy for each of both methods (i.e., the data fusion method and the method using infection data only) (Table 2). In general, the data fusion method tended to produce larger RIs in the combined-countries and between-countries scenarios compared to the within-country scenario. Based on the average RI across scenarios, combining baseline infection data with predictors from other categories usually resulted in positive improvements, with the exception of agricultural predictors (−0.92%). In general, the predictors of biology (8.64%), geography (7.04%), or society (4.89%) provided better improvements compared to the other two categories (infection data around villages (2.13%) and environment (0.47%)).

**Table 2:** The relative improvements (RIs, %) for each scenario obtained using the spatially weighted data fusion method with different predictor configurations (C2–C8), compared to the method with configuration C1.

Combining baseline infection data	Within-country		Combined-countries	Between-countries		Average^1^
With additional predictors	Kenya	Tanzania	Kenya and Tanzania	*Between I*	*Between II*	
Infection around (C2)	0.95	0.00	10.33	6.24	−6.87	2.13
Environment (C3)	2.50	2.29	17.98	−10.57	−9.86	0.47
Agriculture (C4)	0.30	3.40	15.30	−14.54	−9.07	−0.92
Geography (C5)	−0.89	0.43	16.85	17.91	0.88	7.04
Biology (C6)	−0.89	−0.49	16.56	17.18	10.83	8.64
Society (C7)	0.71	−0.43	15.26	15.05	−6.16	4.89
All (C8)	1.37	2.41	16.64	−5.73	11.88	5.31

^1^Average across CV scenarios from columns 2 to 6.

Furthermore, compared to the approach that uses baseline infection data only, the proposed data fusion method typically improved the lower bound of prediction accuracy in most cases based on the lowest accuracy of the 14 prediction models (S4 Table).

### Imbalanced proportions of hotspots and improvement of predictions

This work highlighted the imbalance in the hotspot numbers or proportions (S1 Table). Specifically, the number of hotspots was much higher than that of non-hotspots in Tanzania (e.g., 72% versus 28% from PHS definition I), while in Kenya, the number of hotspots was much smaller than that of non-hotspots (e.g., 35% versus 65% from PHS definition I).

**Fig 3:**
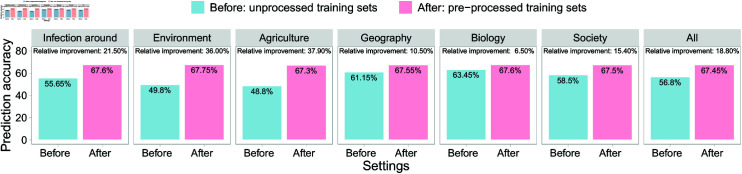
Under PHS definition I, the RIs, in prediction accuracy on unprocessed test sets, obtained from the models trained using pre-processed synthetic data based on the proposed different predictor configurations, compared to models using unprocessed original imbalanced training data, where the best model with the highest prediction accuracy was considered for each method (i.e., y-axis).

Like other areas [41], the performance of the prediction models was heavily affected by the imbalanced distribution of the hotspots. This impact was especially pronounced in the between-countries scenarios, as the proportion of hotspots was completely reversed between Tanzania and Kenya, as analyzed above. As an illustration, we compared the proposed method with the existing method using prediction accuracy presented in S6 Table, where the proposed method was developed using pre-processed training sets, while the reference method was developed using the original unbalanced training sets. For each CV scenario and predictor configuration, we used the highest accuracy to make comparisons by selecting the best model among the fourteen for each method. Based on the highest accuracy of each method, we calculated the RIs of the proposed method. These RIs, along with the highest accuracy, were averaged in the two scenarios between countries, resulting in the ARIs and the average of the highest accuracy for each predictor configuration (Fig 3). Among the seven different predictor configurations, including agricultural predictors contributed to the highest ARI of 37.9%. This was followed by environmental predictors (36%), infection data around villages (21.5%), all predictors (18.8%), societal predictors (15.4%), geographical predictors (10.5%), and biological predictors (6.5%). Detailed RIs achieved by the proposed method are provided in S7 Table, where RIs for a few models are negative, and RIs for most models are positive.

### Importance of each predictor category and each predictor

In general, combining baseline infection data with other predictors improved overall prediction performance of the method that uses baseline infection data only (S4 Fig). Interestingly, including all predictors did not produce the best RI. More specifically, higher average RIs across scenarios were obtained by combining baseline infection data with biology predictors (10%) or geography predictors (8.6%), compared to using all predictors (7.2%).

Furthermore, the importance of predictors was assessed by evaluating their relative effects, quantified as the percentage of variance they explained. Based on CV, we summarized the percentage results along with their standard deviations in S5 Fig. Soil moisture, precipitation, and cropland predictors emerged as the top three most important among all predictors examined in this study. Several environmental predictors (e.g., dew point temperature and irradiation-related variables), socio-geographic and agricultural variables (e.g., buildings, child dependency ratio, population density, permanent water cover fraction), and infection data, also made important contributions to hotspot prediction.

### Scalability of the proposed method for other definitions of persistent hotspots

PHS definition II was used to illustrate the scalability of the proposed method. This definition was considered in recent work [17], in which hotspot predictions in Kenya and Tanzania were also investigated by combining baseline infection data with other datasets. Based on the CV results in S8 Table, this section compared our method with two others based on RIs: (1) the approach that uses only the baseline infection data, and (2) the current results from [17].

The spatially weighted data fusion method achieved improvements in all CV scenarios when compared with the approach using baseline infection data only. For example, when considering the best models for each scenario (within Tanzania, combined-countries, and between-countries) the proposed method achieved a RI of at least 12.2%. Compared to the results provided in S5 Table of [17], the proposed method improved the current results by 2.8% in Kenya and 3.3% in Tanzania. In particular, the previous method for the between-countries scenarios typically produced low accuracy, with the highest accuracy being 31% and 46% in the *between I* and *between II* scenarios, respectively. In contrast, our method for these two scenarios achieved the highest accuracies of 63.1% and 70.2%, respectively, with RIs of 103.5% and 52.6%. However, these comparisons were made in a rough manner, as reproducing their results with the exact same settings is unlikely without their secondary datasets.

### Highlighted models

Another contribution of ours to improving predictions stemmed from the investigation of broader models. In general, the best-performing model in each CV scenario was one of the six newly designed models rather than those from previous work, based on prediction accuracy on the test sets (S6 Fig). Specifically, in these three scenarios (within-country, combined countries, and between-countries), the highest median accuracies were achieved by DyTrees (73.65%), regLogit (72.2%), and LogitGPs (68.1%), respectively. Overall, LogitGPs and DyTrees, which ranked in the top two positions, achieved average accuracies of 70.3% and 70.2%, respectively, while the best among the prior models was the Ensemble model, with an average accuracy of 69.6%. The worst-performing model was a single Tree model, with an average prediction accuracy of 66.1%.

## Discussion

The WHO 2021–2030 Road Map for schistosomiasis set an ambitious public health target: eliminating schistosomiasis as a public health problem by 2030 [18]. However, the formation of hotspots significantly affects preventive chemotherapy effects, thereby hindering the achievement of this goal [23]. Accurate and early identification of hotspots is therefore critical, as it can facilitate the implementation of additional interventions to reduce transmission. To our knowledge, this is the first work to provide insights into developing spatially weighted data fusion techniques and addressing hotspot imbalances to improve the early identification of hotspots. Our primary contributions stemmed from processing data with spatially weighted data fusion and categorization methods, investigating broader and more flexible statistical machine learning models, and addressing hotspot imbalances to improve the performance of prediction models. More specifically, this study offered several novel insights and results for predicting hotspots compared to previous work, highlighted in the following aspects.

First, this work revealed the critical role of schistosomiasis spatial patterns in constructing predictors for prediction models and highlighted spatial variation in prediction accuracy across different geographical regions. Specifically, by analyzing spatial correlations in schistosomiasis and incorporating information from neighboring areas into predictor construction, our method improved the early identification of hotspots compared to approaches that rely solely on baseline infection data. In particular, even without the infection data from the years after the baseline, the proposed method still produced more accurate predictions in most cases compared to previous approaches that used infection data from years 1 and 3 [14] (S3 Text and S9 Table). Furthermore, the CV results showed spatial heterogeneity in prediction performance across subregions (S5 Table). More specifically, local regions bordering larger areas of the lake exhibited lower prediction accuracy than inland subregions. This was likely because villages near broader lake areas experience more complex schistosomiasis transmission dynamics, leading to strong spatial heterogeneity. This made early hotspot identification more difficult.

Second, this study found that simply pooling the datasets often failed to enhance the hotspot predictions, even though variable selection methods had already been used. Specifically, in more than 60% of cases, the best performing models did not use all available data (S3, S8, and S9 Tables). This was likely due to the high complexity of models incorporating all predictors. Despite the use of sparsity techniques intended to mitigate this issue, the estimation bias may be introduced by these techniques, such as LASSO [42]. Therefore, accurate and early identification of hotspots requires more refined methods to construct predictors, such as categorization. Unlike prior studies [17], this work investigated a broader range of data categories to develop predictive models and explored different predictor combinations to reduce the risk of overfitting caused by model complexity. Additionally, it provided a ranking of predictor categories based on their contribution to improving hotspot prediction. These efforts not only enhanced the accuracy of the predictive models, but also supported interpretation of the results and offered deeper insights into the potential drivers of hotspot formation.

Third, this study investigated a range of flexible models capable of capturing intricate correlations, which were not considered in previous studies for hotspot prediction. As noted in earlier work [17], predicting PHSs was particularly challenging. This difficulty stemmed largely from a reciprocal functional relationship between the status of PHSs and baseline prevalence and/or intensity. This suggests that hotspot formation follows a nonlinear dynamic process. To address this, we developed advanced statistical and machine learning models. In particular, LogitGPs demonstrated superior performance over other competing models, since this model can effectively utilize covariance functions to provide a more accurate approximation of the complex relationships between infection data and other predictors, as well as among the predictors themselves [35]. As suggested by the reviewer(s), combining the models into a single stacked ensemble model has the potential to further enhance the performance and generalizability of the proposed method for predicting PHSs.

Fourth, our results suggest that addressing the imbalance of the proportion of hotspots improved the performance of the prediction models, an important aspect that has not been accounted for in previous studies. Imbalanced hotspots were frequently observed in schistosomiasis infection data, negatively affecting the accuracy of prediction models. This study demonstrated that the synthetic oversampling technique can be embedded in the spatially weighted data fusion method to effectively mitigate the impact of imbalances on predictions. This technique was particularly useful in the between-country scenario. This scenario deserves more attention, as it can be used to assess the effectiveness and robustness of methods for handling spatial heterogeneity in infection data and to assess the applicability of prediction methods to other countries. By addressing the imbalanced hotspot issue, the accuracy of the best model in the between-countries scenario was nearly equivalent to that in scenarios within- and combined-countries in some cases (S6, S8, and S9 Tables). However, the room for improvement in the latter two scenarios was relatively limited when the oversampling technique was applied.

Our methodology is scalable. First, the study data are representative in two key respects: (1) *S. mansoni* , one of the six major *Schistosoma* species, is globally distributed, with particularly high prevalence in Africa [9]; and (2) the data from Kenya and Tanzania capture three critical characteristics of schistosomiasis transmission: a complex local geographical environment around the Lake Victoria, the presence of unprotected water sources, and substantial prevalence heterogeneity across subregions [43] (S2 Fig). These reflect a realistic, high spatial heterogeneity setting often encountered in endemic regions [9]. Second, the datasets used in this study and our implementation code are publicly available. The datasets are maintained by authoritative institutions and are regularly updated (S2 Table), which supports the ongoing maintenance and applicability of the proposed method. Furthermore, because these publicly available datasets typically offered global coverage at defined spatial resolutions, our proposed method would be accessible and adaptable for application in other regions beyond the original study area. For applications in other regions, our approach emphasizes the importance of collecting and constructing weighted predictors that are tailored to local geo-social-economic contexts to improve model performance and ensure scalability.

However, this study has several limitations. First, despite efforts to include five predictor categories and collect as many variables as possible within each, some categories contained relatively few predictors. Also, the selection process may have overlooked potentially important categories and predictors, which could introduce bias. Second, key individual-level factors, such as hygiene practices and occupational exposure risks, as well as data on the population distribution of snail intermediate hosts, are unavailable. Including such information could improve hotspot prediction. Third, using a large number of predictors increased the risk of overfitting in statistical and machine learning models. In addition to the predictor categorization mentioned earlier, we employed three strategies to address this issue: (i) during the initial stage of predictor construction, we optimized the candidate predictors by removing those that did not improve prediction accuracy based on CV, resulting in a refined set of inputs for the models; (ii) we incorporated regularization techniques by including models that apply methods such as LASSO and structured Gaussian process assumptions; and (iii) we trained a broad set of 14 models to increase robustness and reduce overfitting risk. However, there is still room for improvement by integrating all models through model ensembling (as mentioned earlier), which could further enhance the scalability and robustness of our prediction framework. This is particularly valuable when applying the approach across heterogeneous geographic regions or multiple countries. Finally, this study focused solely on *S. mansoni*, and hotspot patterns for other *Schistosoma* species may differ [44]. Therefore, additional external validation is necessary before broader implementation of the proposed approach.

## Conclusion

Spatially weighted data fusion enhanced the early identification of hotspots of schistosomiasis. Categorizing predictors refined model development and provided a pathway to investigate which data categories should be prioritized for collection and use in the development of prediction models. The study highlighted the importance of addressing the imbalance of hotspots. The results of the study could support mass drug administration efforts, contribute to the elimination of schistosomiasis, and improve public health.

## Supporting information

Supporting information provided more details, including descriptions of the datasets, cross-validation results, and additional tables and figures.

S1 Fig Study arms of SCORE for schistosomiasis. (TIF)

S2 Fig Baseline infection prevalence across five subregions surrounding Lake Victoria, including two subregions in Tanzania (West and East) and three in Kenya (Northwest, Northeast, and South). The map layers were created using publicly available world map data from Natural Earth, accessed via the R package *rnaturalearth* [24].
(TIF)

S3 Fig Empirical variograms and fitted curves by exponential variogram models. (A) Prevalence in Kenya, (B) Infection prevalence in Tanzania, (C) Infection intensity in Kenya, and (D) Intensity in Tanzania.
(TIF)

S4 Fig Relative improvements (RIs) in prediction accuracy on test sets for the three scenarios from the proposed spatially weighted data fusion method using different predictor categories, compared to the approach using only baseline infection data. (TIF)

S5 Fig The importance of predictors was assessed by examining the relative effects of each predictor, with the effect quantified as the percentage of variance contribution based on multiple Gradient Boosting Machine (GBM) with different hyperparameters. The assessment was repeated 50 times using different training sets, and ten GBM models were run for each evaluation.
(TIF)

S6 Fig Overall performance of each model in each of the three scenarios for predicting PHSs for *S. mansoni*, where the horizontal lines in the boxplots represent the median of accuracy. (TIF)

S1 Table Based on two persistent hotspot (PHS) definitions, the proportion of hotspots (1) and non-hotspots (0) in Kenya and Tanzania. (XLSX)

S2 TableList of predictors used in this study for developing prediction models for early identification of schistosomiasis hotspots.These predictors were collected from SCORE (Schistosomiasis Consortium for the Operational Research and Evaluation) [21], ERA5 (European Centre for Medium-Range Weather Forecasts (ECMWF) Reanalysis v5) [45], GFAS (Global Fire Assimilation System) [46], CGLS (Copernicus Global Land Service) [47], GLAD (Global Land Analysis & Discovery) [48,49], and SDAC (Socioeconomic Data and Applications Center) [50].(XLSX)

S3 Table Detailed accuracy (95% predicted intervals) of each models for predictor configurations C1-C8 under the setting of PHS definition I. (XLSX)

S4 Table Relative improvements (IRs, %) for each scenario obtained using the proposed spatially weighted data fusion methods with different predictor configurations (C2-C8), compared to the method with configuration C1. IRs were obtained from the worst of the 14 models for each method, based on the lowest prediction accuracy for each scenario and predictor configuration.
(XLSX)

S5 Table Detailed accuracy (95% predicted intervals) of each models across subregions of countries for predictor configurations C1-C8 under the setting of PHS definition I. (XLSX)

S6 Table Hotspot prediction accuracy (95% predicted intervals) on test sets obtained using models developed using synthetic sampling training sets, compared to those developed using imbalanced training sets. (XLSX)

S7 Table Relative improvements in hotspot prediction accuracy on test sets obtained using models developed on synthetic sampling training sets, compared to those developed on imbalanced training sets. (XLSX)

S8 Table Detailed accuracy (95% predicted intervals) of each models for predictor configurations C1-C8 under the setting of PHS definition II. (XLSX)

S9 Table Detailed accuracy (95% predicted intervals) of each models for predictor configurations under the setting of PHS definition I, where the previous non-baseline method used the infection data in years 1 and 3 [14], while the proposed data fusion method used the infection data only from year 1 (C2-C8). (XLSX)

S1 Text *Schistosoma mansoni* in Tanzania and Kenya. (DOCX)

S2 Text Categorizing datasets and spatially weighted data fusion. (DOCX)

S3 Text Spatially weighted data fusion methods vs. previous non-baseline methods. (DOCX)
