# Supplementary figures and images for: Identifying key aspects to enhance predictive modeling for early identification of schistosomiasis hotspots to guide mass drug administration
Source: PLoS Negl Trop Dis. 2025 Jul 16;19(7):e0013315. doi: 10.1371/journal.pntd.0013315 (PMC12279088; doi:10.1371/journal.pntd.0013315)

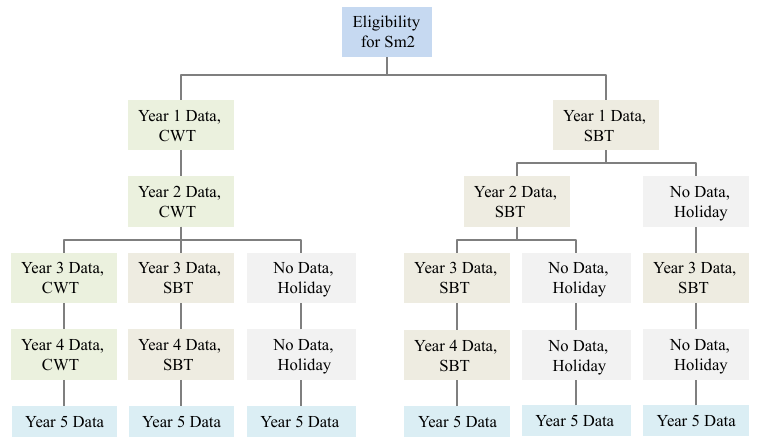

Supplement: S1 Fig — (TIF) [file pntd.0013315.s001.tif]

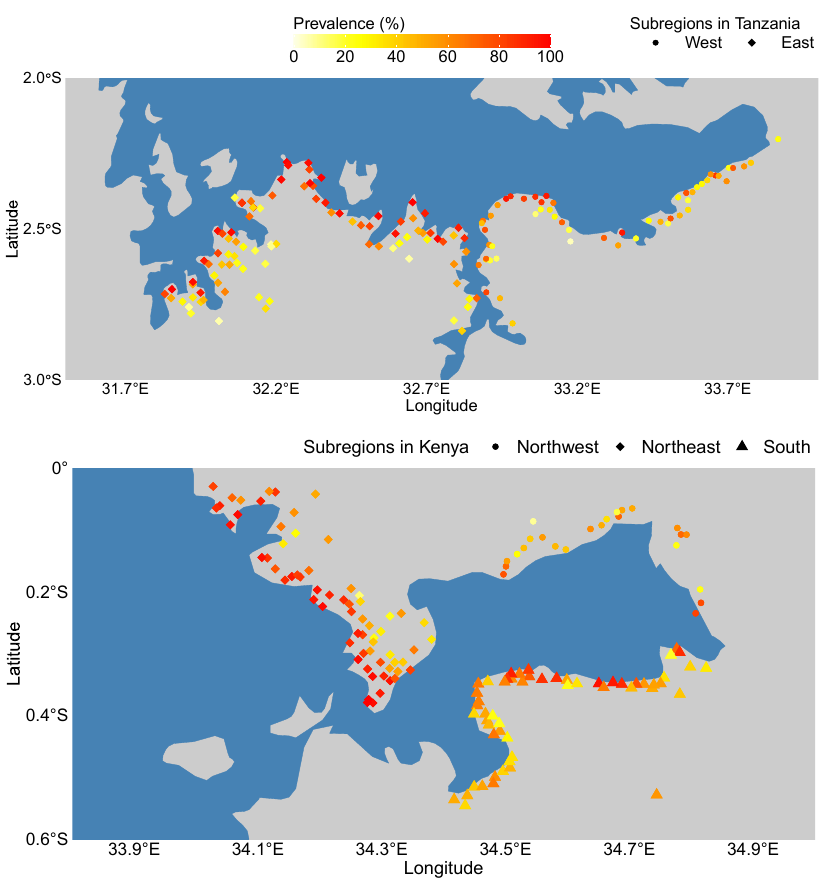

Supplement: S2 Fig — The map layers were created using publicly available world map data from Natural Earth, accessed via the R package rnaturalearth [24]. (TIF) [file pntd.0013315.s002.tif]

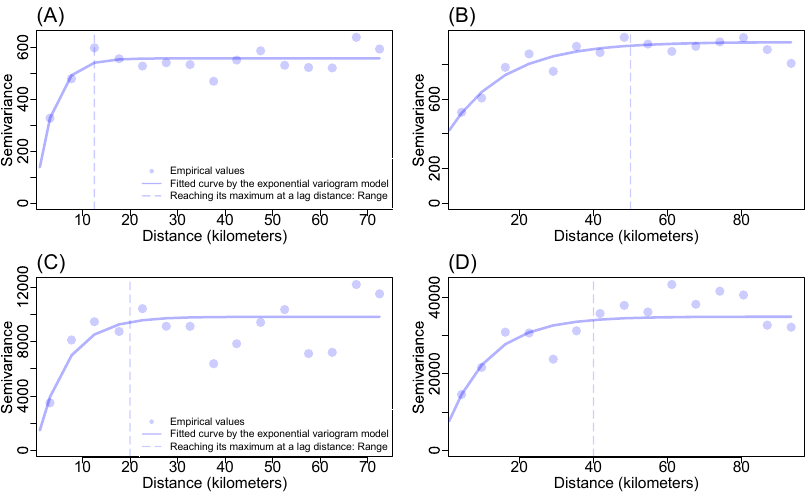

Supplement: S3 Fig — (A) Prevalence in Kenya, (B) Infection prevalence in Tanzania, (C) Infection intensity in Kenya, and (D) Intensity in Tanzania. (TIF) [file pntd.0013315.s003.tif]

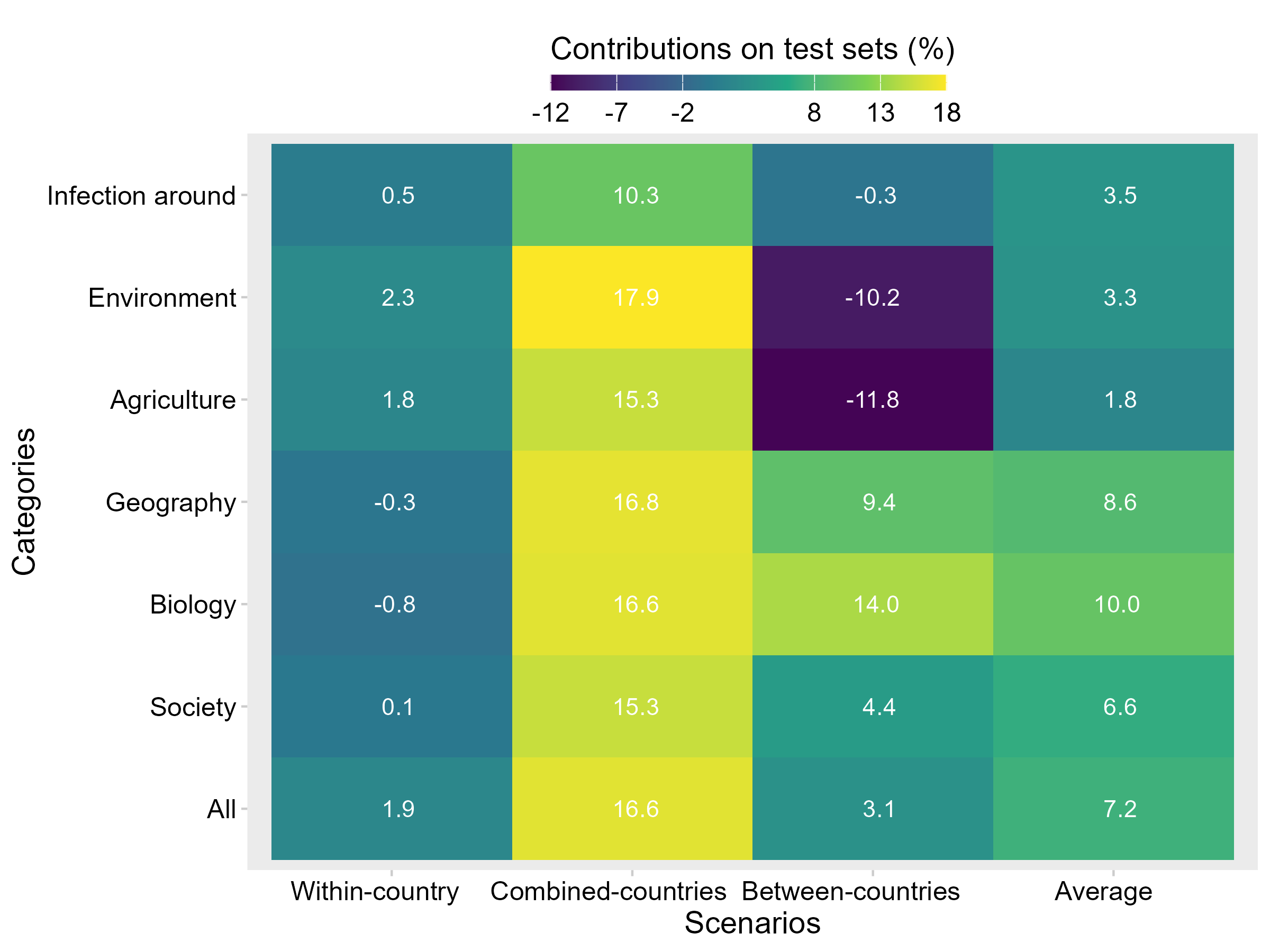

Supplement: S4 Fig — (TIF) [file pntd.0013315.s004.tif]

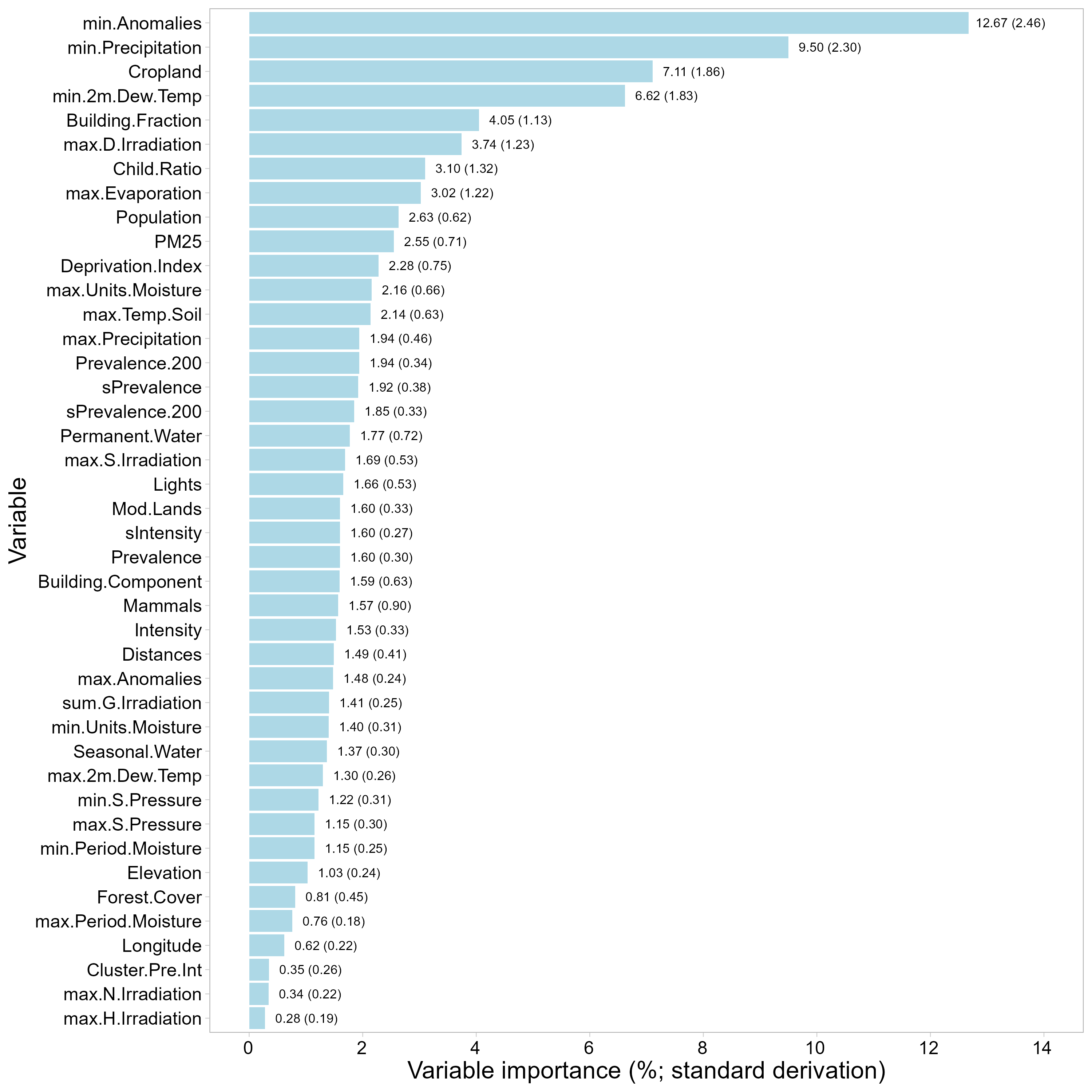

Supplement: S5 Fig — The assessment was repeated 50 times using different training sets, and ten GBM models were run for each evaluation. (TIF) [file pntd.0013315.s005.tif]

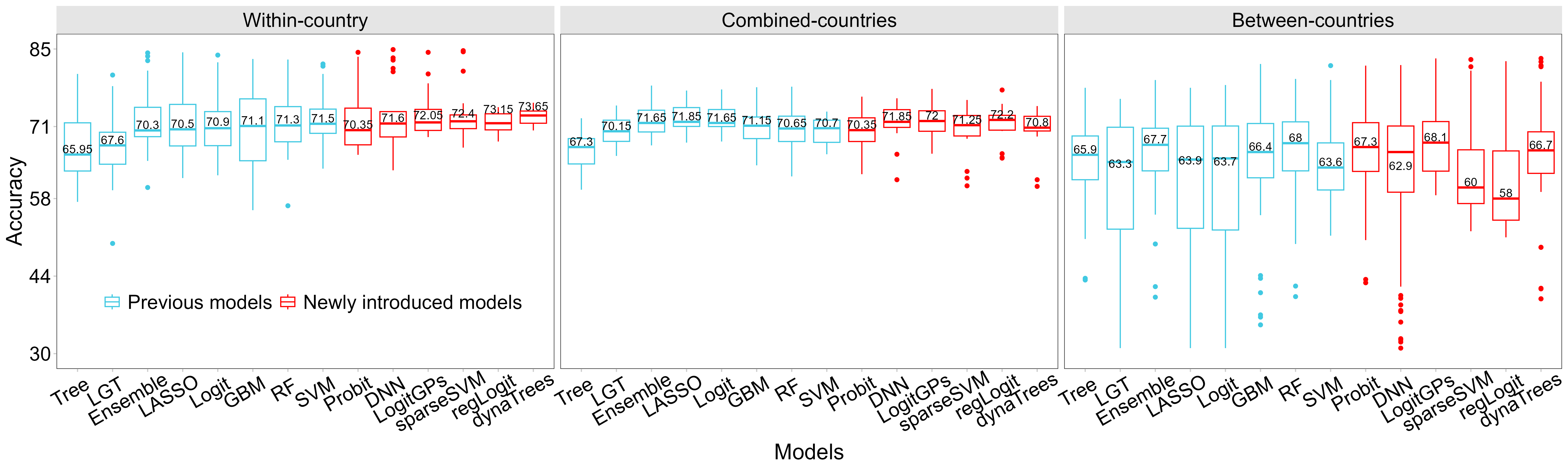

Supplement: S6 Fig — (TIF) [file pntd.0013315.s006.tif]
