# Supplementary material for: Identifying key aspects to enhance predictive modeling for early identification of schistosomiasis hotspots to guide mass drug administration
Source: PLoS Negl Trop Dis. 2025 Jul 16;19(7):e0013315. doi: 10.1371/journal.pntd.0013315 (PMC12279088; doi:10.1371/journal.pntd.0013315)
Supplement: S1 Text — (DOCX) [file pntd.0013315.s016.docx]

***Schistosoma mansoni* in Tanzania and Kenya**

*Schistosoma* infection data for the 295 study villages in Kenya and Tanzania were obtained from the SCORE datasets. The SCORE project conducted a randomized clinical trial from 2011 to 2015, during which the 295 study villages were randomly assigned to six arms and received annual mass drug administration (MDA) with praziquantel (S1 Fig). In Kenya, the infection prevalence ranged from 8.33% to 100%, with a mean of 61.51% and a median of 59.79%. In Tanzania, the minimum prevalence was 4.6%, the average prevalence 54.55%, and the median prevalence 55.32%, all of which were smaller than in Kenya, except for the maximum prevalence of Tanzania, which was the same as that of Kenya. However, the average intensity (49.54 epg (eggs per gram)) and median intensity (89.19 epg) in Kenya were lower than in Tanzania, which were 63 epg and 130.36 epg, respectively. Furthermore, the variation in infection intensity in Kenya was much smaller compared to Tanzania, with the intensity range in the former (3.68-454.86 epg) being much narrower than in the latter (0.96-1138.16 epg). Generally, larger variations can make hotspots more unpredictable, resulting in lower accuracy in predictions (S3 Table).
