# Supplementary material for: Identifying key aspects to enhance predictive modeling for early identification of schistosomiasis hotspots to guide mass drug administration
Source: PLoS Negl Trop Dis. 2025 Jul 16;19(7):e0013315. doi: 10.1371/journal.pntd.0013315 (PMC12279088; doi:10.1371/journal.pntd.0013315)
Supplement: S2 Text — (DOCX) [file pntd.0013315.s017.docx]

**Categorizing datasets and spatially weighted data fusion**

For categorizing datasets, prevalence and intensity were used as baseline disease inputs in prediction models to predict the binary outcome of the PHS status for *S. mansoni*, referred to as the baseline infection data. Furthermore, another predictor, namely the prevalence of infections $\geq200$epg (denoted by prevalence.200), was also incorporated into the baseline infection data. In addition, 41 other predictors were employed to improve the accuracy of the models in hotspot predictions. To ensure the interpretability of the hotspot prediction results and gain deeper insight into the factors that potentially drive hotspot formation, a knowledge-driven category formation method was used to classify the datasets. This approach resulted in a categorization of predictors into six distinct categories: *infection data around villages*, *environment,* *agriculture*, *geography*, *biology*, and *society*.

In spatially weighted data fusion for predictor construction, this study not only integrated spatial information from neighboring areas within a specified distance (threshold) but also simultaneously filtered out redundant noise beyond that threshold. This can be achieved using the spTIDW method, and the threshold can be determined by examining a spatial correlation range based on empirical variograms (S3 Fig). Using a 20 km threshold in spTIDW, three predictors (prevalence, intensity, and prevalence.200) of the baseline infection data were spatially weighted to build predictors of *infection data around villages*. To capture similar patterns of *S. mansoni* in different villages, the K-Means clustering method with K = 2 was used for prevalence and intensity in the baseline year, resulting in a binary variable. Then, this variable was assigned to the category of *infection data around villages* as a predictor. Using a threshold of 50 km, spTIDW was applied to construct other spatially weighted predictors from the remaining categories. In contrast to the annual scale of the SCORE datasets, the weighted predictors from other categories were typically on a smaller scale, such as monthly. To align these with infection data on a yearly scale, the weighted predictors were calculated as mean, maximum, minimum, or sum over time. S2 Table for more details of these predictors.

In particular, the spTIDW approach employed in our work helps mitigate these issues because spTIDW for each village uses multiple values of the original variable within the local spatial region to generate a weighted candidate predictor. This approach offers two key advantages: (1) The generated candidate predictors are spatially local smoothing predictors, making them robust to outliers; and (2) When the original variable contains missing data, the corresponding missing values from surrounding areas are excluded from the weighted calculation in spTIDW, thereby avoiding the need for imputation. In particular, we used cross-validation procedures to evaluate multiple distance thresholds (6 km, 12 km, 20 km, 40 km, and 50 km) for *infection data around villages* based on empirical variogram model exploration. For predictors from other categories, we adopted a more conservative 50 km threshold based on empirical experience, as computational constraints limited our ability to perform extensive cross-validation. After completing the spTIDW procedure, we optimized the candidate predictors by removing those that did not improve prediction accuracy, resulting in a refined set of inputs for the models.
