# Supplementary material for: Identifying key aspects to enhance predictive modeling for early identification of schistosomiasis hotspots to guide mass drug administration
Source: PLoS Negl Trop Dis. 2025 Jul 16;19(7):e0013315. doi: 10.1371/journal.pntd.0013315 (PMC12279088; doi:10.1371/journal.pntd.0013315)
Supplement: S3 Text — (DOCX) [file pntd.0013315.s018.docx]

**Spatially weighted data fusion methods vs. previous non-baseline methods**

S9 Table shows the accuracy of each model for the predictor configurations, where the models were developed based on four of the six arms. This is because two of the six arms lacked infection data in the third year (S1 Fig), and infection data from the third year were necessary inputs for the previous non-baseline method required. The proposed data fusion method improved the hotspot prediction results of the previous non-baseline method on test sets for Tanzania, combined-countries, and between-countries scenarios.
